# *Porphyromonas gingivalis* predicts local recurrence after endoscopic submucosal dissection of early esophageal squamous cell carcinoma or precancerous lesion

**DOI:** 10.1186/s12885-022-10469-8

**Published:** 2023-01-13

**Authors:** She-Gan Gao, Zhi-Peng Qi, Yi-Jun Qi, Ying-Yong Hou, Yi-Wen Liu, Meng-Xiang Li, Bing Li, Di Sun, Qiang Shi, Shi-Lun Cai, Ping-Hong Zhou, Yun-Shi Zhong

**Affiliations:** 1 State Key Laboratory of Esophageal Cancer Prevention & Treatment, Henan Key Laboratory of Microbiome and Esophageal Cancer Prevention and Treatment, Henan Key Laboratory of Cancer Epigenetics, Cancer Hospital, The First Affiliated Hospital, College of Clinical Medicine, Henan University of Science and Technology, 471003 Luoyang, China; 2 Endoscopy Center, Zhongshan Hospital of Fudan University, 200032 Shanghai, China; 3 Endoscopy Research Institute of Fudan University, 200032 Shanghai, China; 4 Department of Pathology, Zhongshan Hospital of Fudan University, 200032 Shanghai, China

**Keywords:** Esophageal squamous cell carcinoma, *Porphyromonas gingivalis*, Endoscopic submucosal dissection, Local recurrence

## Abstract

**Background:**

*Porphyromonas gingivalis* plays an oncogenic role in development and progression of esophageal squamous cell carcinoma (ESCC). However, the impact of *P. gingivalis* on local recurrence of early ESCC or precancerous lesion after ESD treatment remains unknown. The present study aimed to evaluate the impact of *P. gingivalis* on local recurrence after ESD treatment of early ESCC or high-grade dysplasia (HGD).

**Methods:**

The amount of *P. gingivalis* was assessed by immunohistochemistry in 205 patients with early ESCC or HGD. Univariate and multivariate Cox regression analyses were performed to determine the effect of *P. gingivalis* on local recurrence. Propensity score matching analysis was performed to reduce the imbalance of baseline characteristics. A nomogram integrating significant prognostic factors was built for local recurrence prediction.

**Results:**

The amount of *P. gingivalis* increased significantly in neoplasms that invaded up to muscularis mucosa and submucosa compared with lesions confined to epithelium or lamina propria. Overabundance of *P. gingivalis* was positively associated with invasion depth, post-ESD stricture and local recurrence. Univariate and multivariate Cox regression analyses revealed that *P. gingivalis*, longitudinal length of lesion and lymphovascular invasion were independent predictors for post-ESD recurrence. A nomogram comprising *P. gingivalis*, lymphovascular involvement, and lesion length performed well for prediction of post-ESD local recurrence with the concordance indices of 0.72 (95%CI, 0.62 to 0.80), 0.72 (95%CI, 0.63 to 0.80), and 0.74 (95%CI, 0.65 to 0.83) in the validation cohort, the entire cohort, and the subcohort after PSM, respectively.

**Conclusion:**

*P. gingivalis* overabundance is a risk factor and a potential predictor for local recurrence of early ESCC or HGD after ESD treatment. Thus, clearance of *P. gingivalis* represents an attractive strategy for prognosis improvement and for prevention of ESCC.

## Introduction

Esophageal squamous cell carcinoma (ESCC) histologically represents more than 90% of esophageal cancer in China [1, 2], which is the fourth most common cancer and the fourth leading cause of cancer-related mortality of the country [3]. In China, the overall 5-year survival rate is about 10% due to the highly aggressiveness of ESCC whereas the long-term outcome is greatly improved if ESCC diagnosed in the early stages [4–6]. Therefore, it is of paramount importance to identify early neoplasia confined to esophagus, which represents the most cost-effective approach to cure ESCC. With the widespread application of esophagogastroscopy, an increasing number of patients are diagnosed in the early stages of ESCC [7–9], which comprises neoplasia limited to m1 (intraepithelia), m2 (invasion of the lamina propria), m3 (invasion of the muscularis mucosa), and superficial submucosa (invasion of submucosa < 500 μm), without any lymphatic or vascular invasion.

At present, these localized diseases can be cured by endoscopic resection (ER) including endoscopic mucosal resection (EMR) and endoscopic submucosal dissection (ESD), surgical resection, and chemoradiotherapy [9–14]. Considering the mortality and morbidity associated with esophagectomy, ER is the treatment of choice for the early-stage ESCC with an almost null risk of lymph node metastasis or for high surgical risk patients with ESCC, especially in elderly patients with concurrent illnesses [7, 10, 11, 15, 16]. Recently, ESD has been recommended to replace EMR because larger and deeper lesions of esophageal cancers can be completely resected by ESD with a higher probability of cure than that of EMR [7, 17–20]. However, it is challenging to identify patients in the early stage of ESCC without lymph node involvement. Numerous retrospective studies demonstrate that lymph node invasion correlates with the depth of tumor invasion, tumor histology and differentiation, and lymphatic or vascular invasion [7]. In the case of invasion depth of lesions, it is reported that lymph node metastases were observed in 0–4.0% of cancers confined to the epithelium and lamina propria, in 0-22.2% of muscularis mucosa cancers and in 25.9–53.8% of submucosal cancers [10, 11, 21–25]. However, the depth of invasion can only be accurately examined once histological examination of the resected specimen is performed. On the other hand, a subset of early-stage ESCC patients after ER develops metastasis or recurrence regardless of invasion depth [10, 11]. Thus, it is more pressing to identify markers related to post-ESD recurrence.

A growing body of evidence indicates a potential causative role of microbial dysbiosis in the etiology of ESCC [26–28]. *Porphyromonas gingivalis (P. gingivalis)*, a key-stone periodontal pathogen, is enriched in ESCC compared to adjacent mucosal tissues, and overabundance of *P. gingivalis* was associated with poor-differentiation, metastasis, and shorter survival of ESCC patients [29, 30]. Furthermore, serum immunoglobulin G and A antibodies against *P. gingivalis* have the potential for diagnosis of ESCC including early-stage ESCC, suggesting a tumorigenic role of *P. gingivalis* in the development and progression of ESCC [31]. However, the potential effect of *P. gingivalis* on clinical outcome of ER has not been reported in literature. Herein, we examined whether *P. gingivalis* impacts the outcome of early esophageal epithelial lesions after ESD.

## Materials and methods

### Patient selection

This study enrolled 205 consecutive patients with early ESCC or high-grade dysplasia (HGD) from the endoscopy center of Zhongshan Hospital of Fudan University between August 2008 and September 2013. The early ESCC comprised m2 ESCC, m3 ESCC, and submucosal ESCC (sm1 ESCC) invading into the submucosal layer up to 200 μm. Inclusion criteria were histologically confirmed HGD or early ESCC, complete resection, absence of residual lesions, no metastasis, alive till the end of the follow-up period, availability of hematoxycilin and eosin slides, and submucosal invasion less than 200 μm. Exclusion criteria were residual lesions after ESD, adenocarcinoma or other diseases before ESD treatment, submucosal invasion more than 200 μm, esophagectomy performed immediately after ESD, esophagogastric junction lesions, and unwilling to commit to complete the follow-up. All data including demography, histopathology, clinical parameters, and follow-up data were recorded in the electronic medical database.

This study was approved by the medical ethics committee of The First Affiliated Hospital of Henan University of Science and Technology, in accordance with the Declaration of Helsinki. Informed consent was waived because this study used retrospective and anonymized individual data.

### ESD procedure

All ESD procedure was performed according to our previous report [32]. The senior endoscopists performed ESD with patients under general anesthesia. The ESD was conducted using the hook-knife and isolated-tip knife, or dual knife (Olympus). After ESD, antibiotics (celaclor or cefuroxime), hemocoagulase injections (ethamsylate or P-aminomethybenzoic acid) and omeprazole (40 mg/day) were routinely administered. Patients were put on a soft diet on the 3rd day after ESD treatment unless serious symptoms or complications occurred. The hospitalization was generally 2 to 3 days after ESD treatment.

### Histopathological examination

Resected tissue specimens were immobilized onto a Styrofoam plate using thin needles along their edges, fixed in 10% formalin, paraffin-embedded, cut into 5 μm sections and subjected to hematoxylin and eosin staining. All sections were examined microscopically for histological type, depth of invasion, and lymph vascular invasion. Complete resection by ESD was defined as the complete removal of an iodine-unstained lesion or the marking dots placed around the lesion with tumor-free lateral and basal margins. The histology, classification and differentiation were assessed according to the World Health Organization classification [15].

### Immunohistochemistry and assessment

Immunohistochemistry (IHC) of *P. gingivalis* was performed as described previously [29]. Abundance of *P. gingivalis* in formalin-fixed, paraffin-embeded sections of early lesions of ESCC was assigned a score using a semiquantitative 4-category grading system incorporating staining intensity and percentage of positively stained cells. Youden Index was used for identification of optimal cut-off point to predict local recurrence after ESD.

### RNA in situ hybridization

The deparaffinized and rehydrated sections were incubated with citrate buffer (10 nmol/L, pH 6) at 100 °C for 15 min, 10 µg/mL protease (Sigma-Aldrich, St. Louis, MO) at 40 °C for 30 min, *P. gingivalis-*specific probes in hybridization buffer A (6 × SSC) for 3 h, preamplifier hybridization buffer B for 30 min, amplifier hybridization buffer B at 40 °C for 30 min, label probe for 15 min, and developed using RED, followed by counterstaining with hematoxylin.

### Data collection and follow-up

Clinicopathological and endoscopic features including age, gender, disease location, size, tumor type, and histological findings were collected for analysis. After ESD, patients enrolled in our follow-up program were examined with endoscopy at 1, 2, 6, and 12 months in the first year and annually thereafter. Lugol’s staining and biopsy were performed when the previous resection site appeared suspicious for residual or recurrent lesions. Serum squamous cell carcinoma antigen measurement, abdominal ultrasonography and computed tomography were performed in patients if necessary. Follow-up was defined as the time between the date of ESD and January 31, 2018. Local recurrence was defined as metachronous esophageal lesions occurred three months after ESD. Time to local recurrence was calculated from the date of ESD to the time of diagnosis of local recurrence.

### Statistical analysis

Statistical analyses were conducted using SPSS 24.0 for Windows (SPSS, Chicago, IL). The differences between categorical variables were compared using the χ^2^ test or Fisher’s exact test. Local recurrence rates for different variables were depicted using the Kaplan-Meier method and were compared using the log-rank test. Receiver operating characteristic (ROC) curve was generated using the sensitivity and specificity for each predictor of local recurrence within 5 years after ESD. The ROC curves were used to select the optimal cutoff value for *P. gingivalis* immunostaining score. The univariate and multivariate Cox proportional hazards regression models were used to identify predicative factors for local recurrence. A nomogram was constructed base on the results of multivariate analysis using the R version 4.1.0. The performance of the nomogram was evaluated by concordance index (C-index) and calibration plots. To build a nomogram model, all 205 patients were randomly allocated to the training subgroup (143 patients) and the validation subgroup (62 patients) at a ratio of 7:3 using “sample” in R language [33, 34].

Propensity score matching (PSM) analysis [33, 34] was performed to reduce the imbalance between groups with high- and low-abundance of *P. gingivalis* based on invasion depth and post-ESD stricture. A nearest-neighbor matching algorithm was utilized for PSM analysis using a multivariable logistic regression model with a tolerated difference between propensity scores less than 20% of the propensity score SD. Propensity scores were calculated for patient matching in a 1:2 ratio with a caliper of 0.01 without replacement.

## Results

### Clinicopathological characteristics of patients

A total of 205 patients were recruited in this study. Table 1 shows the clinicopathological characteristics of patients. The lesions comprised 79 (38.5%) HGDs, 46 (22.4%) m2 ESCCs, 73 (35.6%) m3 ESCCs, and 7 (3.4%) sm1 ESCCs. The median follow-up time of these patients was 64 months (range, 5 to 110 months). During follow-up, 14.6% (30 of 205) of the patients developed local recurrence, and 29.3% (60 of 205) of the patients developed post-ESD stricture. The 1-year, 3-year and 5-year recurrence-free survival rates were 97.6%, 90.2%, and 85.9%, respectively.

**Table 1:** Demographic and clinicopathological characteristics of the entire cohort of patients

Variable	Entire cohort *(n* = 205) (%)
Age	
< 60	85 (41.5)
≥ 60	120 (58.5)
Gender	
Male	156 (76.1)
Female	49 (23.9)
Tobacco smoker	
Yes	107 (52.2)
No	98 (47.8)
Alcohol drinker	
Yes	109 (53.2)
No	96 (46.8)
Histological grade	
Well-differentiation	9 (4.4)
Moderate-differentiation	186 (90.7)
Poor-differentiation	10 (4.9)
Longitudinal length of lesion(cm)	
<3	98 (47.8)
≥ 3	107 (52.2)
Circumferential range	
<3/4	189 (92.2)
>3/4	16 (7.8)
Invasion depth	
HGD & m1 ESCC	125 (60.9)
m2 & sm1 ESCC	80 (39.0)
Lymphovascular invasion	
Yes	9 (4.4)
No	196 (95.6)
Post-ESD stricture	
Yes	60 (29.3)
No	145 (70.7)
Budding (mm)	
>5	13 (6.3)
≤ 5	192 (93.7)
*P. gingivalis*	
High	69 (33.7)
Low	136 (66.3)
Local recurrence	
Yes	30 (14.6)
No	175 (85.4)

*HGD *High grade dysplasia, *m2 ESCC *ESCC invading the lamina propria, *m3 ESCC *ESCC invading the mucularis mucosa, *sm1 ESCC *ESCC invading the submucosal layer less than 200 μm

### Enrichment of *P. gingivalis* in esophageal mucosa

**Fig. 1:**
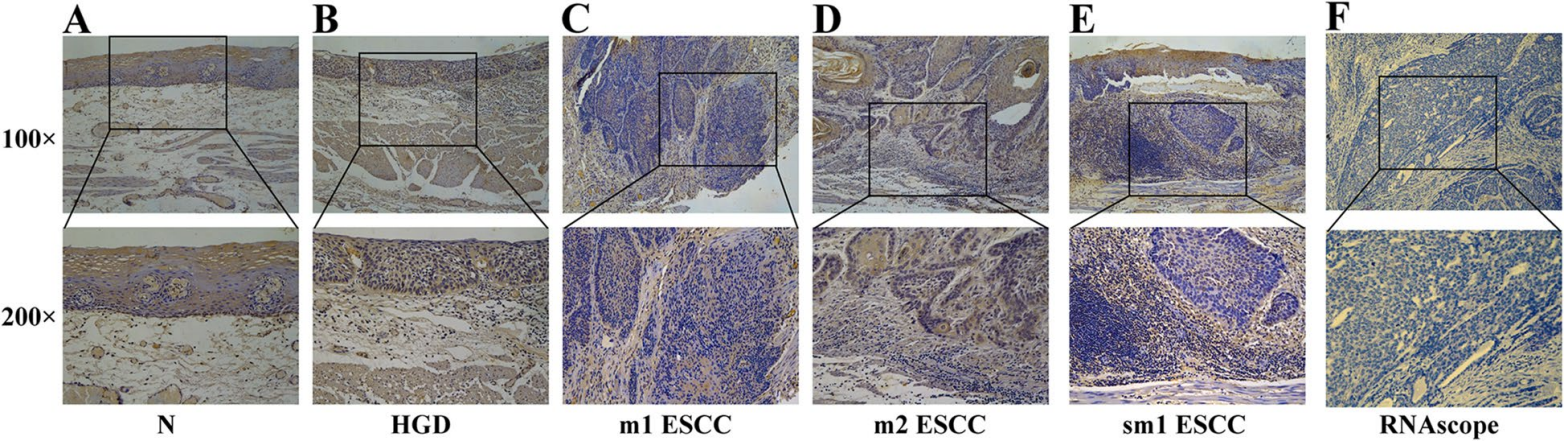
Representative images of immunohistochemical staining and RNA in situ hybridization of *P. gingivalis* in esophageal tissues. **A**-**E** Immunohistochemical staining of *P. gingivalis* in normal epithelium and different lesions of esophagus. **F** RNA in situ hybridization (RNAscope) detection of *P. gingivalis* in ESCC tissues. N, Normal esophageal epithelium; HGD, High grade dysplasia; m2 ESCC, ESCC invading the lamina propria; m3 ESCC, ESCC invading the mucularis mucosa; sm1 ESCC, invading the submucosal layer less than 200 μm; RNAscope, RNA in situ hybridization

The presence of *P. gingivalis* was not scarce in normal esophageal mucosa and the amount of *P. gingivalis* localized in superficial cells was higher than that in basal cells, suggesting that superficial epithelial cells may prevent *P. gingivalis* from invasion into deeper layer cells (Fig. 1A). In dysplastic epithelial cells, immunostaining of *P. gingivalis* was diffuse but more pervasive than that in normal mucosa (Fig. 1B). In cancerous cells, however, heterogeneous immunostaining of *P. gingivalis* was detected in ESCC of m2, m3 and sm1 (Fig. 1C-E). Not surprisingly, *P. gingivalis* was located in tumor stromal tissues as well. Notably, the frequency of *P. gingivalis* enrichment elevated significantly during the transition from m2 to m3/sm1 ESCC (28.0% vs. 42.5%, *P* < 0.05). In addition, RNA in situ hybridization (designated as RNAscope) revealed a similar distribution of *P. gingivalis* in the cytoplasm of cancer cells and stroma as well (Fig. 1F).

### Associations between *P. gingivalis* and clinicopathological characteristics

We next examined the associations between *P. gingivalis* and clinicopathological characteristics. The overabundance of *P. gingivalis* was associated with invasion depth (*P* = 0.035), post-ESD stricture (*P* = 0.034), and local recurrence (*P* = 0.006), but not with the other features (Table 2). These results indicate that *P. gingivalis* may potentially promote aggressiveness of early ESCC.

### Local recurrence after ESD

**Fig. 2:**
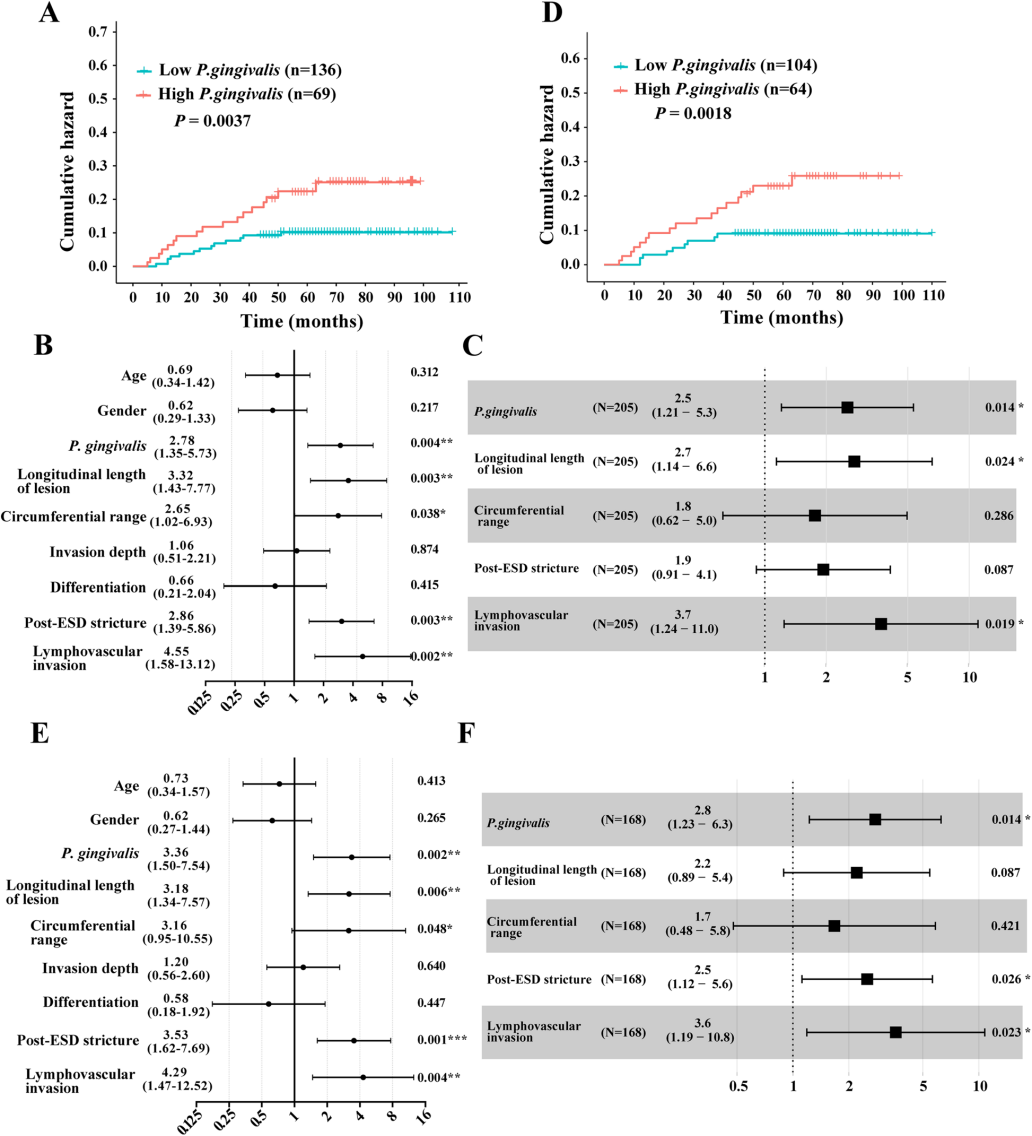
*P. gingivalis* predicts local recurrence after ESD. **A** The cumulative local recurrence rates of patients with low- and high-levels of *P. gingivalis.* **B** Univariate associations of *P. gingivalis* and clinicopathological characteristics with local recurrence. **C** Multivariate associations of *P. gingivalis* and clinicopathological characteristics with local recurrence. **D** The cumulative local recurrence rates of patients with low- and high-levels of *P. gingivalis* after propensity score matching. **E** Univariate associations of *P. gingivalis* and clinicopathological characteristics with local recurrence after propensity score matching. **F** Multivariate associations of *P. gingivalis* and clinicopathological characteristics with local recurrence after propensity score matching

According to the cut-off score of *P. gingivalis* immunostaining of 3, 66.3% of the patients (136 of 205) were assigned to the low-*P. gingivalis* group and 33.7% of the patients (69 of 205) to the high-*P. gingivalis* group, respectively. High amounts of *P. gingivalis* were strongly associated with higher rates of local recurrence compared with low levels of *P. gingivalis*. The cumulative 5-year local recurrence rate was 23.2% among patients with high-*P. gingivalis* (*n* = 69) and 9.6% among patients with low-*P. gingivalis* (*n* = 136), with a hazard ratio (HR) of 2.78 (95% CI, 1.35 to 5.73, *P* = 0.004, Fig. 2A).

### Independent prognostic predictors

Univariate and multivariate Cox regression analyses were performed to identify independent predictors for post-ESD local recurrence. Univariate analyses revealed that *P. gingivalis* overabundance (HR, 2.78; 95% CI, 1.35 to 5.73; *P* = 0.004) and aggressive histopathological features including longitudinal length of lesion (HR, 3.32; 95% CI, 1.43 to 7.77; *P* = 0.003), circumferential range (HR, 2.65; 95% CI, 1.02 to 6.93, *P* = 0.038), post-ESD stricture (HR, 2.86; 95% CI, 1.39 to 5.86; *P* = 0.003), and lymphovascular invasion (HR, 4.55; 95% CI, 1.58 to 13.12; *P* = 0.002) were positively and significantly associated with higher local recurrence (Fig. 2B). Multivariate analyses demonstrated that *P. gingivalis* overabundance (HR, 2.5; 95% CI, 1.21 to 5.3; *P* = 0.014), longitudinal length of lesion (HR, 2.7; 95% CI, 1.14 to 6.6; *P* = 0.024), and lymphovascular invasion (HR, 3.7; 95% CI, 1.24 to 11.0; *P* = 0.019) remained independent predictors for local recurrence (Fig. 2C).

### Propensity score matching analysis

As there were significant differences between the high- and low-abundance of *P. gingivalis* groups with regards to invasion depth and post-ESD stricture, we applied a PSM analysis to minimize these differences in a 1:2 ratio. After matching, 64 and 104 patients were included in the high- and low-*P. gingivalis* groups, respectively. The characteristics of invasion depth and post-ESD stricture were evenly distributed between these groups after matching (Table 2). Again, the high abundance of *P. gingivalis* rendered a higher risk of local recurrence after ESD treatment compared with low abundance of *P. gingivalis.* The cumulative 5-year local recurrence rate was significantly higher in patients with high-*P. gingivalis* (*n* = 64; 26.6%, 95% CI, 15.4–37.7%) as compared with patients with low-*P. gingivalis* (*n* = 104; 8.7%, 95% CI, 3.2–14.1%, Fig. 2D). The results of the univariate analysis resemble the results before PSM (Fig. 2E). Multivariate analyses revealed that *P. gingivalis* overabundance (HR, 2.80; 95% CI, 1.23 to 6.30; *P* = 0.014), post-ESD stricture (HR, 2.50; 95% CI, 1.12 to 5.60; *P* = 0.026), and lymphovascular invasion (HR, 3.60; 95% CI, 1.19 to 10.80; *P* = 0.023) were independent predictors for local recurrence after PSM (Fig. 2F).

**Table 2:** Associations between *P. gingivalis* abundance and clinicopathological characteristics

Variable	Entire cohort (*n* = 205)			Matched cohort (*n* = 168)		
	*P. gingivalis*			*P. gingivalis*		
	Low (*n* = 136)(%)	High (*n* = 69)(%)	*P* value	Low (*n* = 104)(%)	High (*n* = 64)(%)	*P* value
Age						
< 60	57 (41.9)	28 (40.6)	0.882	38 (36.5)	27 (42.2)	0.516
≥ 60	79 (58.1)	41 (59.4)		66 (63.5)	37 (57.8)	
Gender						
Male	103 (50.2)	53 (25.9)	0.864	81 (77.9)	50 (78.1)	0.95
Female	33 (16.1)	16 (7.8)		23 (22.1)	14 (21.9)	
Tobacco smoker						
Yes	69 (50.7)	38 (55.1)	0.657	63 (60.6)	36 (56.2)	0.63
No	67 (49.3)	31 (44.9)		41 (39.4)	28 (43.8)	
Alcohol drinker						
Yes	70 (51.5)	39 (56.5)	0.554	61 (58.7)	36 (56.2)	0.872
No	66 (48.5)	30 (43.5)		43 (41.3)	28 (43.8)	
Histological grade						
Well-differentiation	7 (5.1)	2 (2.9)	0.585	6 (5.8)	1 (1.6)	0.318
Moderate-differentiation	121 (89.0)	65 (94.2)		91 (87.5)	61 (95.3)	
Poor-differentiation	8 (5.9)	2 (2.9)		7 (6.7)	2 (3.1)	
Longitudinal length of lesion(cm)						
< 3	64 (47.1)	34 (49.3)	0.77	56 (53.8)	30 (46.9)	0.428
≥ 3	72 (52.9)	35 (50.7)		48 (46.2)	34 (53.1)	
Circumferential range						
< 3/4	124 (91.2)	65 (94.2)	0.586	100 (96.2)	60 (93.8)	0.481
> 3/4	12 (8.8)	4 (5.8)		4 (3.8)	4 (6.2)	
Invasion depth						
HGD & m1 ESCC	90 (66.2)	35 (50.7)	0.035	64 (61.5)	33 (51.6)	0.26
m2 & sm1 ESCC	46 (33.8)	34 (49.3)		40 (38.5)	31 (48.4)	
Lymphovascular invasion						
Yes	5 (3.7)	4 (5.8)	0.489	5 (4.8)	4 (6.2)	0.732
No	131 (96.3)	65 (94.2)		99 (95.2)	60 (93.8)	
Post-ESD stricture						
Yes	33 (24.3)	27 (39.1)	0.034	27 (26.0)	22 (34.4)	0.295
No	103 (75.7)	42 (60.9)		77 (74.0)	42 (65.6)	
Budding (mm)						
≤ 5	128 (94.1)	64 (92.8)	0.765	97 (93.3)	60 (93.8)	1
>5	8 (5.9)	5 (7.2)		7 (6.7)	4 (6.2)	
Local recurrence						
Yes	13 (9.6)	17 (24.6)	0.006	9 (8.7)	17 (26.6)	0.004
No	123 (90.4)	52 (75.4)		95 (91.3)	47 (73.4)	

*HGD* High grade dysplasia, *m2 ESCC *ESCC invading the lamina propria, *m3 ESCC *ESCC invading the mucularis mucosa, *sm1 ESCC *ESCC invading the submucosal layer less than 200 μm

### Building a prognostic nomogram for post-ESD local recurrence

**Fig. 3:**
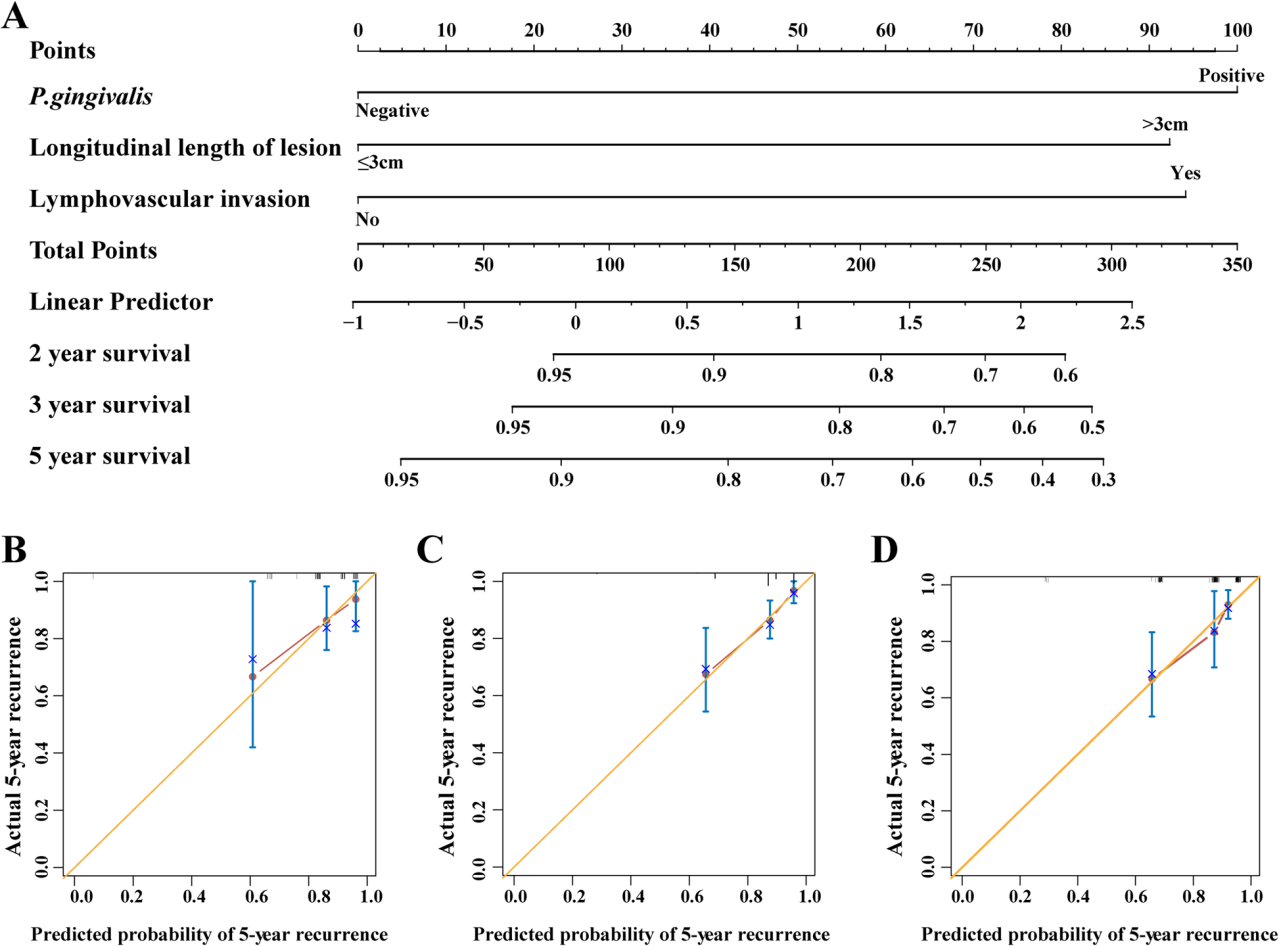
Construction and validation of nomogram for predicting local recurrence after ESD. **A** Nomogram for predicting 2-, 3-, and 5-year local recurrence for patients after ESD in training cohort. **B**-**D** Calibration curves for the nomogram to predict 5-year local recurrence in the validation cohort (**B**), the entire cohort (**C**), and the subcohort after propensity score matching (**D**). The yellow line represents the ideal nomogram, and the red line represents the observed nomogram

To develop a clinically applicable tool, we built a predicative model that integrated the three significant prognostic factors using nomogram (Fig. 3A). As shown in the nomogram, *P. gingivalis* contributed largest to local recurrence, followed by lymphovascular invasion and lesion length. The calibration plot demonstrated an optimal agreement between the nomogram prediction and actual observation for the probability of local recurrence at 5 years in the validation cohort, the entire cohort and the subcohort after propensity score matching. The C-indices for local recurrence prediction of nomogram score were 0.72 (95% CI, 0.62 to 0.80), 0.72 (95%CI, 0.63 to 0.80), and 0.74 (95%CI, 0.65 to 0.83), respectively (Fig. 3B-D). Then, we compared the predictive power for post-ESD local recurrence between nomogram score and *P. gingivalis*, longitudinal length of lesion and lymphovascular invasion. The C-indices by *P. gingivalis*, longitudinal length of lesion, and lymphovascular invasion were inferior to that of nomogram without statistical significance in the entire cohort (0.62, 0.63 and 0.55, respectively).

## Discussion

In this study, we have demonstrated that enrichment of *P. gingivalis* not only occurred in early-stage ESCC but also positively correlated with the severity of lesions of early ESCC during progression from mild to severe lesions. An increased amount of *P. gingivalis* significantly correlated with invasion depth and post-ESD stricture. Moreover, overabundance of *P. gingivalis* was strongly associated with a higher rate of local recurrence and remained an independent predictor for local recurrence, which was reinforced after PSM. In the present study, a nomogram based on three independent prognostic factors was developed and showed optimal prediction of local recurrence after ESD treatment. To our knowledge, this is the first study to report the effects of *P. gingivalis* on local recurrence of early-stage lesions of ESCC after ESD treatment in patients with early ESCC or precancerous lesions. Thus, enrichment of *P. gingivalis* is a potential surrogate marker for prediction of aggressive progression of ESCC.

The known risk factors identified in high-risk areas for ESCC in China comprise nutritional deficiencies [35], family history of ESCC [36], alcohol drinking, cigarette smoking [3, 37], nitrosamine-rich or mycotoxin-contaminated foods, and low socioeconomic status [38–40]. In a prospective cohort study involved 28 868 subjects in northern China, the first few tooth loss in younger subjects was associated with a significant elevated risk for developing cancers from esophagus and stomach [41]. The salivary overall microbial diversity was decreased in ESCC patients compared with dysplasia patients and healthy controls [27, 28]. Higher abundance of periodontal pathogen *P. gingivalis* was associated with an increased ESCC risk [26]. Recently, we reported that overabundance of *P. gingivalis* was significantly correlated with aggressive features of advanced-stage ESCC [29]. In keeping with these results, *P. gingivalis* overabundance was observed in 30.4% (24/79) of precancerous lesions HGD. Notably, the prevalence and abundance of *P. gingivalis* were significantly increased in ESCC of m3 and sm1 compared with HGD, m1 and m2 ESCC. Taken together, these results suggest that *P. gingivalis* is linked to the initiation and progression of ESCC.

At present, endoscopic screening of high-risk population in high-incidence areas for ESCC has been widely accepted as an optimal strategy for the secondary prevention of ESCC in China [42, 43]. With the emergence of high-resolution and high-definition endoscopy, detailed delineation of mucosal features enables detection of subtle lesions, which consequently results in the increased detection of early neoplasms [9]. Importantly, endoscopic resection is now becoming the standard care for the treatment of esophageal squamous cell dysplasia and early-stage ESCC, and produces less mortality and morbidity, and equivalent survival times compared with esophagectomy [9, 10]. However, endoscopic resection for early ESCC has a relatively high recurrence rate [44, 45] and many factors including tumor volume and degree of infiltration have been identified as the risk factors for the recurrence [17]. In our study, we demonstrated that classification of patients with early-stage ESCC by *P. gingivalis* abundance can substantially differentiate the high-risk from low-risk patients for local recurrence. Alternatively, multiple iodine-unstained lesions correlated with a higher risk of local recurrence after ER of early ESCC [11]. In line with this, we identified longitudinal length of lesion as an independent risk factor for local recurrence. In addition, we found that *P. gingivalis* was the largest contributor to post-ESD local recurrence, suggesting the tumorigenic role of *P. gingivalis* in occurrence of ESCC. Collectively, our findings suggest that an enrichment of *P. gingivalis* is a potential surrogate marker for metachronous recurrence after ESD, which warrants future clinical studies to clarify.

Given that nomogram has been widely used for prognostic predication of cancer patients, we constructed a nomogram based on three independent factors of local recurrence including *P. gingivalis*, lymphovascular invasion, and lesion length, which performed well for prediction of local recurrence post-ESD therapy. Further prospective studies are needed to test its prognostic value.

It is acknowledged that the present study has limitations. This was a single-institute retrospective study, which inevitably introduces selection and information biases. A multi-center, prospective study with a larger sample size is warranted to confirm the tumor-promoting effects of *P. gingivalis* in earl-stage ESCC. Second, semi-quantification of *P. gingivalis* by IHC is not an accurate and sensitive method compared to Q-PCR. Third, various storage duration of resected tissue specimen by ESD may compromise the quality of *P. gingivalis* for gauge. Fourth, one single time point specimen was collected after ESD to measure the amount of *P. gingivalis*, which may underestimate the effect of *P. gingivalis* on esophageal cancer recurrence during the 5-year follow-up.

## Conclusion

The present study demonstrates that *P. gingivalis* enrichment occurs in esophageal precancerous lesions and early-stage ESCC, and an overabundance of *P. gingivalis* is associated with local recurrence after ESD treatment of patients with early ESCC. In addition, we developed a nomogram for post-ESD recurrence prediction. Thus, our findings suggest that clearance of *P. gingivalis* in clinical management of ESCC patients, in particular for post-ESD patients, represents an attractive strategy to prevent local recurrence and improve the prognosis of early stage ESCC. In high-risk subjects predisposing for ESCC, *P. gingivalis-*specific treatment in oral cavity opens a novel approach for ESCC prevention.

## Data Availability

All data generated or analyzed during this study are included in this published article.

## Abbreviations

ESCC: Esophageal squamous cell carcinoma
HGD: High-grade dysplasia
ER: Endoscopic resection
EMR: Endoscopic mucosal resection
ESD: Endoscopic submucosal dissection
m2 ESCC: ESCC invading the lamina propria
m3 ESCC: ESCC invading the mucularis mucosa
sm1 ESCC: ESCC invading the submucosal layer less than 200 μm
IHC: Immunohistochemistry
ROC: Receiver operating characteristic
C-index: Concordance index
PSM: Propensity score matching

## References

[1] Xu Y, Yu X, Chen Q, Mao W (2012). Neoadjuvant versus adjuvant treatment: which one is better for resectable esophageal squamous cell carcinoma?. World J Surg Oncol.

[2] Torre LA, Bray F, Siegel RL, Ferlay J, Lortet-Tieulent J, Jemal A (2015). Global cancer statistics, 2012. Cancer J Clin.

[3] Lin Y, Totsuka Y, He Y, Kikuchi S, Qiao Y, Ueda J, Wei W, Inoue M, Tanaka H (2013). Epidemiology of esophageal cancer in Japan and China. J Epidemiol.

[4] Zeng H, Zheng R, Guo Y, Zhang S, Zou X, Wang N, Zhang L, Tang J, Chen J, Wei K (2015). Cancer survival in China, 2003–2005: a population-based study. Int J Cancer.

[5] Wang GQ, Jiao GG, Chang FB, Fang WH, Song JX, Lu N, Lin DM, Xie YQ, Yang L (2004). Long-term results of operation for 420 patients with early squamous cell esophageal carcinoma discovered by screening. Ann Thorac Surg.

[6] Pennathur A, Gibson MK, Jobe BA, Luketich JD (2013). Oesophageal carcinoma. Lancet (London England).

[7] Pimentel-Nunes P, Dinis-Ribeiro M, Ponchon T, Repici A, Vieth M, De Ceglie A, Amato A, Berr F, Bhandari P, Bialek A (2015). Endoscopic submucosal dissection: european Society of Gastrointestinal Endoscopy (ESGE) Guideline. Endoscopy.

[8] Zeng H, Ran X, An L, Zheng R, Zhang S, Ji JS, Zhang Y, Chen W, Wei W, He J (2021). Disparities in stage at diagnosis for five common cancers in China: a multicentre, hospital-based, observational study. The Lancet Public health.

[9] Mannath J, Ragunath K (2016). Role of endoscopy in early oesophageal cancer. Nat reviews Gastroenterol Hepatol.

[10] Jiang D, Li X, Wang H, Xu C, Li X, Sujie A, Zeng H, Hou Y, Zhong Y (2017). A retrospective study of endoscopic resection for 368 patients with early esophageal squamous cell carcinoma or precancerous lesions. Surg Endosc.

[11] Yamashina T, Ishihara R, Nagai K, Matsuura N, Matsui F, Ito T, Fujii M, Yamamoto S, Hanaoka N, Takeuchi Y (2013). Long-term outcome and metastatic risk after endoscopic resection of superficial esophageal squamous cell carcinoma. Am J Gastroenterol.

[12] Crumley AB, Going JJ, McEwan K, McKernan M, Abela JE, Shearer CJ, Stanley AJ, Stuart RC (2011). Endoscopic mucosal resection for gastroesophageal cancer in a U.K. population. Long-term follow-up of a consecutive series. Surg Endosc.

[13] Higuchi K, Tanabe S, Azuma M, Katada C, Sasaki T, Ishido K, Naruke A, Katada N, Koizumi W (2013). A phase II study of endoscopic submucosal dissection for superficial esophageal neoplasms (KDOG 0901). Gastrointest Endosc.

[14] Yamada M, Oda I, Nonaka S, Suzuki H, Yoshinaga S, Taniguchi H, Sekine S, Kushima R, Saito Y, Gotoda T (2013). Long-term outcome of endoscopic resection of superficial adenocarcinoma of the esophagogastric junction. Endoscopy.

[15] Bhatt A, Abe S, Kumaravel A, Vargo J, Saito Y (2015). Indications and techniques for endoscopic submucosal dissection. Am J Gastroenterol.

[16] Kuwano H, Nishimura Y, Oyama T, Kato H, Kitagawa Y, Kusano M, Shimada H, Takiuchi H, Toh Y, Doki Y (2015). Guidelines for Diagnosis and Treatment of Carcinoma of the Esophagus April 2012 edited by the Japan Esophageal Society. Esophagus.

[17] Wang Z, Lu H, Wu L, Yuan B, Liu J, Shi H, Wang F (2016). Long-term outcomes of endoscopic multiband mucosectomy for early esophageal squamous cell neoplasia: a retrospective, single-center study. Gastrointest Endosc.

[18] Ishihara R, Iishi H, Uedo N, Takeuchi Y, Yamamoto S, Yamada T, Masuda E, Higashino K, Kato M, Narahara H (2008). Comparison of EMR and endoscopic submucosal dissection for en bloc resection of early esophageal cancers in Japan. Gastrointest Endosc.

[19] Cao Y, Liao C, Tan A, Gao Y, Mo Z, Gao F (2009). Meta-analysis of endoscopic submucosal dissection versus endoscopic mucosal resection for tumors of the gastrointestinal tract. Endoscopy.

[20] Evans JA, Early DS, Chandraskhara V, Chathadi KV, Fanelli RD, Fisher DA, Foley KQ, Hwang JH, Jue TL, Pasha SF (2013). The role of endoscopy in the assessment and treatment of esophageal cancer. Gastrointest Endosc.

[21] Akutsu Y, Uesato M, Shuto K, Kono T, Hoshino I, Horibe D, Sazuka T, Takeshita N, Maruyama T, Isozaki Y (2013). The overall prevalence of metastasis in T1 esophageal squamous cell carcinoma: a retrospective analysis of 295 patients. Ann Surg.

[22] Araki K, Ohno S, Egashira A, Saeki H, Kawaguchi H, Sugimachi K (2002). Pathologic features of superficial esophageal squamous cell carcinoma with lymph node and distal metastasis. Cancer.

[23] Eguchi T, Nakanishi Y, Shimoda T, Iwasaki M, Igaki H, Tachimori Y, Kato H, Yamaguchi H, Saito D, Umemura S. Histopathological criteria for additional treatment after endoscopic mucosal resection for esophageal cancer: analysis of 464 surgically resected cases. Modern Pathology. 2006;19(3):475–480. 10.1038/modpathol.3800557 16444191

[24] Tajima Y, Nakanishi Y, Ochiai A, Tachimori Y, Kato H, Watanabe H, Yamaguchi H, Yoshimura K, Kusano M, Shimoda T (2000). Histopathologic findings predicting lymph node metastasis and prognosis of patients with superficial esophageal carcinoma: analysis of 240 surgically resected tumors. Cancer.

[25] Kim DU, Lee JH, Min BH, Shim SG, Chang DK, Kim YH, Rhee PL, Kim JJ, Rhee JC, Kim KM (2008). Risk factors of lymph node metastasis in T1 esophageal squamous cell carcinoma. J Gastroenterol Hepatol.

[26] Peters BA, Wu J, Pei Z, Yang L, Purdue MP, Freedman ND, Jacobs EJ, Gapstur SM, Hayes RB, Ahn J (2017). Oral Microbiome Composition reflects prospective risk for esophageal cancers. Cancer Res.

[27] Yu G, Gail MH, Shi J, Klepac-Ceraj V, Paster BJ, Dye BA, Wang GQ, Wei WQ, Fan JH, Qiao YL (2014). Association between upper digestive tract microbiota and cancer-predisposing states in the esophagus and stomach. Cancer Epidemiol Biomarkers Prevent..

[28] Chen X, Winckler B, Lu M, Cheng H, Yuan Z, Yang Y, Jin L, Ye W (2015). Oral microbiota and risk for esophageal squamous cell carcinoma in a high-risk area of China. PLoS ONE.

[29] Gao S, Li S, Ma Z, Liang S, Shan T, Zhang M, Zhu X, Zhang P, Liu G, Zhou F (2016). Presence of Porphyromonas gingivalis in esophagus and its association with the clinicopathological characteristics and survival in patients with esophageal cancer. Infect agents cancer.

[30] Gao S, Liu Y, Duan X, Liu K, Mohammed M, Gu Z, Ren J, Yakoumatos L, Yuan X, Lu L (2021). Porphyromonas gingivalis infection exacerbates oesophageal cancer and promotes resistance to neoadjuvant chemotherapy. Br J Cancer.

[31] Gao SG, Yang JQ, Ma ZK, Yuan X, Zhao C, Wang GC, Wei H, Feng XS, Qi YJ (2018). Preoperative serum immunoglobulin G and a antibodies to Porphyromonas gingivalis are potential serum biomarkers for the diagnosis and prognosis of esophageal squamous cell carcinoma. BMC Cancer.

[32] Shi Q, Ju H, Yao LQ, Zhou PH, Xu MD, Chen T, Zhou JM, Chen TY, Zhong YS (2014). Risk factors for postoperative stricture after endoscopic submucosal dissection for superficial esophageal carcinoma. Endoscopy.

[33] Zhou W, Jin W, Wang D, Lu C, Xu X, Zhang R, Kuang T, Zhou Y, Wu W, Jin D (2019). Laparoscopic versus open pancreaticoduodenectomy for pancreatic ductal adenocarcinoma: a propensity score matching analysis. Cancer Commun (London England).

[34] Bettinger D, Pinato DJ, Schultheiss M, Sharma R, Rimassa L, Pressiani T, Burlone ME, Pirisi M, Kudo M, Park JW (2019). Stereotactic body Radiation Therapy as an alternative treatment for patients with Hepatocellular Carcinoma compared to Sorafenib: a propensity score analysis. Liver cancer.

[35] Guo W, Blot WJ, Li JY, Taylor PR, Liu BQ, Wang W, Wu YP, Zheng W, Dawsey SM, Li B (1994). A nested case-control study of oesophageal and stomach cancers in the Linxian nutrition intervention trial. Int J Epidemiol.

[36] Wei WQ, Abnet CC, Lu N, Roth MJ, Wang GQ, Dye BA, Dong ZW, Taylor PR, Albert P, Qiao YL (2005). Risk factors for oesophageal squamous dysplasia in adult inhabitants of a high risk region of China. Gut.

[37] Wu M, Zhao JK, Zhang ZF, Han RQ, Yang J, Zhou JY, Wang XS, Zhang XF, Liu AM, van’. t Veer P et al. Smoking and alcohol drinking increased the risk of esophageal cancer among Chinese men but not women in a high-risk population. Cancer Causes Control. 2011; 22(4):649–657. 10.1007/s10552-011-9737-4 PMC3059761 21321789

[38] Roth MJ, Strickland KL, Wang GQ, Rothman N, Greenberg A, Dawsey SM (1998). High levels of carcinogenic polycyclic aromatic hydrocarbons present within food from Linxian, China may contribute to that region’s high incidence of oesophageal cancer. Eur J cancer (Oxford England: 1990).

[39] Lu SH, Ohshima H, Fu HM, Tian Y, Li FM, Blettner M, Wahrendorf J, Bartsch H (1986). Urinary excretion of N-nitrosamino acids and nitrate by inhabitants of high- and low-risk areas for esophageal cancer in Northern China: endogenous formation of nitrosoproline and its inhibition by vitamin C. Cancer Res.

[40] Tran GD, Sun XD, Abnet CC, Fan JH, Dawsey SM, Dong ZW, Mark SD, Qiao YL, Taylor PR (2005). Prospective study of risk factors for esophageal and gastric cancers in the Linxian general population trial cohort in China. Int J Cancer.

[41] Abnet CC, Qiao YL, Mark SD, Dong ZW, Taylor PR, Dawsey SM (2001). Prospective study of tooth loss and incident esophageal and gastric cancers in China. Cancer causes & control: CCC.

[42] He Z, Liu Z, Liu M, Guo C, Xu R, Li F, et al. Efficacy of endoscopic screening for esophageal cancer in China (ESECC): design and preliminary results of a population-based randomised controlled trial. Gut. 2018;70(2):251–60. 10.1136/gutjnl-2017-315520 29306867

[43] Chen R, Liu Y, Song G, Li B, Zhao D, Hua Z, Wang X, Li J, Hao C, Zhang L (2021). Effectiveness of one-time endoscopic screening programme in prevention of upper gastrointestinal cancer in China: a multicentre population-based cohort study. Gut.

[44] Ono S, Fujishiro M, Niimi K, Goto O, Kodashima S, Yamamichi N, Omata M (2009). Long-term outcomes of endoscopic submucosal dissection for superficial esophageal squamous cell neoplasms. Gastrointest Endosc.

[45] Esaki M, Matsumoto T, Hirakawa K, Nakamura S, Umeno J, Koga H, Yao T, Iida M (2007). Risk factors for local recurrence of superficial esophageal cancer after treatment by endoscopic mucosal resection. Endoscopy.

